# Pegylated interferon 2a and ruxolitinib induce a high rate of oral complications among patients with myeloproliferative neoplasms

**DOI:** 10.1002/jha2.15

**Published:** 2020-06-01

**Authors:** Marie Orliaguet, Sylvie Boisramé, Jean‐Richard Eveillard, Brigitte Pan‐Petesch, Marie‐Anne Couturier, Vincent Rebière, Gaelle Guillerm, Marie‐Caroline Le Bousse‐Kerdiles, Laurent Misery, Eric Lippert, Jean‐Christophe Ianotto

**Affiliations:** ^1^ Département de Chirurgie Orale CHRU de Brest Brest France; ^2^ Service d'Hématologie Clinique, Institut de Cancéro‐Hématologie CHRU de Brest Brest France; ^3^ EA3878, Groupe d'Etude des Thromboses de Bretagne Occidentale, GETBO CHRU de Brest Brest France; ^4^ UMR‐S‐MD 1197, Institut André Lwoff Université Paris XI Villejuif France; ^5^ France Intergroupe des Néoplasies Myéloprolifératives (FIM); ^6^ Service de Dermatologie CHRU de Brest Brest France; ^7^ Laboratoire d'Hématologie CHRU de Brest Brest France; ^8^ INSERM, UMR 1078 Université de Brest Brest France

Clinical manifestations in myeloproliferative neoplasms (MPN) include blood hyperviscosity (polycythaemia vera [PV], essential thrombocythaemia [ET]), resulting in higher thrombosis risk, possibly organomegaly (chronic myelogenous leukaemia (CML)), primary/secondary myelofibrosis (PMF/SMF) and in some instance cytopenia‐related manifestations (PMF, SMF and/or treatment related) [1, 2]. Besides the relatively common occurrence of hydroxycarbamide‐induced aphtosis, clinical manifestations of MPN rarely affect the oral sphere [3]. However, we noticed that patients treated for MPN often seek dentist care for various reasons. In order to confirm this, we undertook a questionnaire‐based assessment of oral care need in our cohort of MPN patients.

Over a period of 3 months, 203 patients consulting for MPN follow‐up were given a short questionnaire asking whether they had to seek dental care in the previous 2 years and the reason (gingival abscess, tooth crack or tooth loss). All patients included in this study were 18 years‐old or older, all of them were followed for a minimum time of 2 years. Some of them were treated for their MPN. All patients with complete denture were excluded from the analyses. All patients gave their nonopposition to participate in the local OBENE registry (NCT02897297). A control cohort of 133 patients consulting in the dermatology department was given the same questionnaire.

The principal characteristics of the MPN population are presented in **Table S1**. Note that 87.2% of the patients had Ph‐negative MPN (n = 177) (40.9% with ET, 30.5% with PV, 15.8% with MF and 12.8% with CML). The sex ratio was 1:1. The median follow‐up time since diagnosis was 7.4 years. At the time of the questionnaire, 93.1% of the patients received a cytoreductive therapy, mainly with hydroxycarbamide (42.9%), pegylated interferon α2a (Peg‐Inf) (15.9%) or ruxolitinib (14.3%). All 26 patients with CML were under BCR‐ABL1 kinase inhibitors (TKI). The distribution of drugs is presented in **Table S2**. The control population had a median age of 65 years (*P* = .42) and a sex ratio of 1.1 (*P* = .6).

Overall, 39 patients (19.2%) had sought oral cares, five of which had multiple cares, resulting in 44 events. The most common reasons were spontaneous tooth cracks (n = 19, 43.2%), spontaneous tooth losses (n = 13, 29.6%) and gingival abscesses (n = 12, 27.3%). In the control population, only 13 patients (9.8%) sought oral care (for 14 events in total) (OR = 2.19 [1.087; 4.676], *P* = .02). Needs for oral care were differently distributed among MPN: 35.7, 22.6, 13.3 and 7.7% in patients with MF, PV, ET or CML, respectively (*P* = 0.01).

There was no difference according to age, gender or driver mutation for Phi‐negative MPN. Buccodental complications have rarely been described in MPN. However, MPN treatments may impact on oral health. For instance, hydroxycarbamide is known to induce mouth ulcers, local squamous cell carcinoma and senescence of dental follicle stem cells [3].

We then analyzed the impact of ongoing treatment on oral care needs. These were more frequent in patients receiving ruxolitinib (51.7%) or Peg‐Ifn (30%), and then hydroxycarbamide (16%) and TKI (7.7%). None were recorded in patients receiving anagrelide or pipobroman (*P* = .000056). Patients treated with ruxolitinib or Peg‐Ifn represented 28.1% of the total cohort but 59% of patients having sought oral cares and all five cases of multiple cares. Treatment with Ruxolitinib or Peg‐Ifn increased the risk of needing oral care by 5.52 times (*P* = .0005) and 2.22 times (*P* = .11), respectively, compared to hydroxycarbamide. Patients receiving ruxolitinib mostly suffered from tooth cracks (64.3% of cares), whereas those treated with Peg‐Ifn had higher incidence of gingival abscesses (77.8% of cares) (**Table S3**). Curves of incidence of tooth cares and gingival abscesses are presented in Figure 1.

**FIGURE 1:**
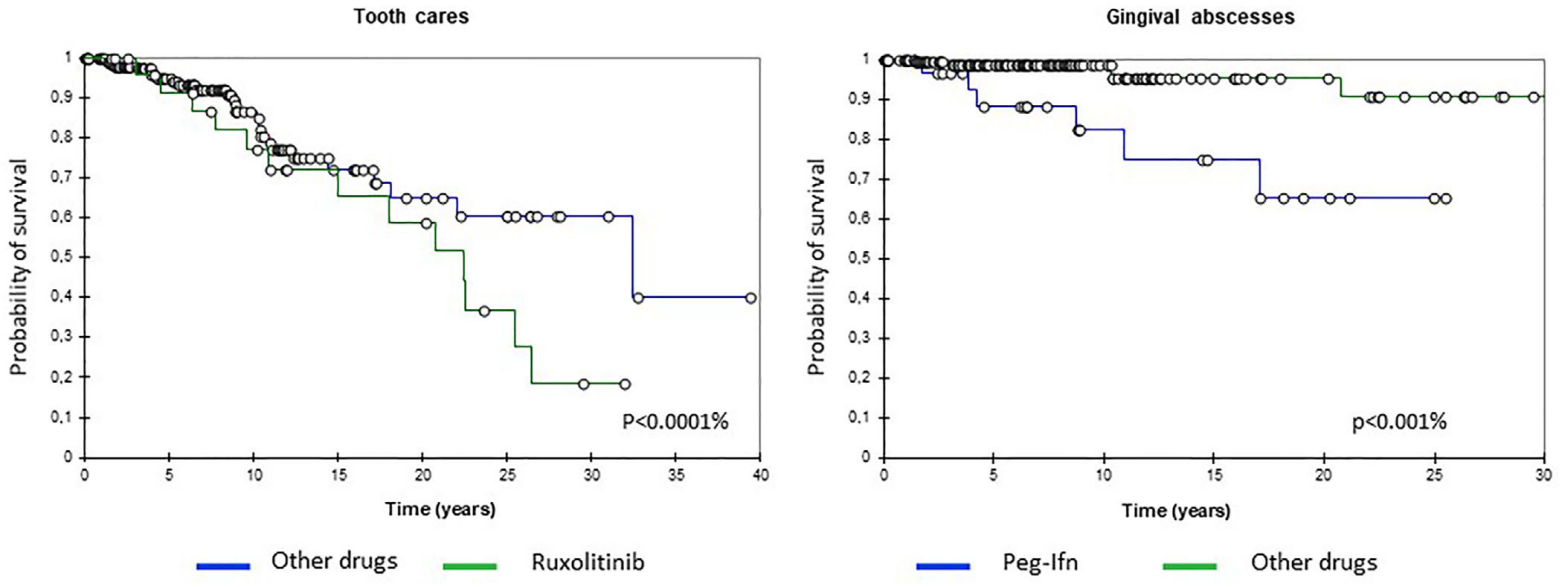
Kaplan‐Meier curves of incidence of tooth cares and gingival abscesses according to MPN treatments

In a multivariate analysis including all parameters described above, treatment with Peg‐Ifn (OR = 11.4) or ruxolitinib (OR = 14.77) are the most significant parameters associated with need for oral care (*P* < .0001 each). Peg‐Ifn use was highly associated with gingival abscess (OR = 5.8, *P* = 0.004) and to a lesser degree to tooth loss (OR = 3.93, *P* = 0.053), whereas there was a nonsignificant trend toward association of ruxolitinib with tooth cracks (OR = 2.34, *P* = 0.097) (Table 1).

**TABLE 1:** Univariate and multivariate analyses about oral cares observed in our population

	Oral cares		Gingival abscesses		Tooth cracks		Tooth losses	
	*P*	OR	*P*	OR	*P*	OR	*P*	OR
Univariate analysis								
Women	.9	NC	.63	NC	.76	NC	.67	NC
≥60 years	.38	NC	.13	0.41 [0.106;1.72]	.33	NC	1	NC
FU ≥ 5 years	1	NC	.52	NC	1	NC	.77	NC
Ph‐neg MPN	.179	3.16 [0.727;28.8]	.88	NC	.66	NC	1	NC
JAK2 status	.71	NC	.62	NC	.53	NC	.46	NC
<2 lines	.23	0.68 [0.36;1.28]	.79	NC	.9	NC	.14	0.45 [0.46;1.29]
Peg‐Ifn	.11	2.22 [0.73;6.587]	**.00039**	**23.54 [2.799;1103.42]**	.7	NC	.24	NC
Ruxolitinib	**.0005**	**5.52 [1.930;16.359]**	.82	NC	**.04**	**2.73 [1.04;7.19]**	.26	2,16 [0.411;10.028]
Multivariate analysis								
Peg‐Ifn	**<.0001**	**11.4 [3.65;35.63]**	**.004**	**5.8 [1.75;19.25]**	NC	NC	.053	3.93 [0.98;15.75]
Ruxolitinib	**<.0001**	**14.77 [4.05;53.79]**	NC	NC	.097	2.34 [0.86;6.4]	NC	NC
<2 lines	**<.0001**	**0.093 [0; 0.30]**	.063	0.307 [0.088;1.067]	NC	NC	**.046**	**0.227 |0;0.98]**

The bold values were those significant.

Peg‐Ifn has mainly been studied in hepatitis C patients, associated with ribavirin and shown to induce gingival abscesses (4%) [4]. In our study, up to 30% of the patients treated with Peg‐Ifn have needed oral care, particularly gingival abscesses (*P* = .004, OR = 3.54). This may be explained by the decrease in circulating neutrophils often observed in Peg‐Ifn‐treated patients [5]. This high risk is of importance in the context of increased prescription of interferon, especially with the recent development of Peg‐Ifn α2b (Ropeg‐interferon), now approved in first line for PV patients [6].

Ruxolitinib has been shown to favour infections and skin cancers [7]. In the COMFORT‐I trial, 11 of 266 (4.1%) patients suffered from gingival abscesses: seven of 155 cases were identified in the ruxolitinib arm, four of 111 were found after the switch from placebo to ruxolitinib while no case has been reported among 151 patients in the placebo arm [8]. No other information regarding dental complications is available from the published clinical trials. In our cohort, ruxolitinib was associated with significantly a higher risk of global oral complications like tooth cracks. The implication of JAK kinases in dentine and enamel formation may be part of the explanation [9]. Indeed, various kinds of stem cells including mesenchymal stromal cells, mostly derived from the neural crest, reside in the pulp or periodontal region and play a role in tissue maintenance and repair. Neural crest stem cells depend on several biochemical pathways for their differentiation and functions, including JAK1/Stat3. One can assume that JAK1/2 inhibitors could compromise their function, thus resulting in dental frailty [10].

Poor oral health can seriously affect patient's quality of life in many ways (pain, infection, modification of food intake, need for additional treatments, etc.) and have economic consequences. We show here that MPN patients have a high rate of oral complications, mainly related to treatments. Larger cohorts should be studied to more precisely decipher the causes and mechanisms of these. Also, having haematologist and dentist work in close relationship through cross‐disciplinary consultations should help identify and treat promptly these complications.

## AUTHOR CONTRIBUTIONS

Jean‐Christophe Ianotto and Eric Lippert designed the study. Jean‐Christophe Ianotto and Jean‐Richard Eveillard made the statistics. Jean‐Christophe Ianotto and Eric Lippert wrote the paper. Marie Orliaguet and Sylvie Boisramé saw the patients for oral cares. Brigitte Pan‐Petesch, Marie‐Anne Couturier, Vincent Rebière, Gaelle Guillerm, Eric Lippert and Jean‐Christophe Ianotto saw patients with MPN. Marie‐Caroline Le Bousse‐Kerdiles helped in the results/discussion section. Laurent Misery saw patients with dermatological problems. All the authors validated the final version of the article.

## CONFLICT OF INTEREST

The authors declared no conflict of interest.

## Supporting information

SUPPORTING INFORMATION
