# Supplementary material for: Pegylated interferon 2a and ruxolitinib induce a high rate of oral complications among patients with myeloproliferative neoplasms
Source: EJHaem. 2020 Jun 1;1(1):350–2. doi: 10.1002/jha2.15 (PMC9176112; doi:10.1002/jha2.15)
Supplement: Supplementary file 1 — SUPPORTING INFORMATION [file JHA2-1-350-s001.docx]

**Supplementary Table 1.** **Characteristics of the population.**

| **Parameters** |  | **n (%)** |
| --- | --- | --- |
| Cohort |  | 203 |
| Median age (y) | | 68.5 |
| Females |  | 50.2% |
| Median Follow-up (y) | | 7.4 |
| Neoplasms | Essential thrombocythaemia | 83 (40.9) |
|  | Polycythaemia vera | 62 (30.5) |
|  | Myelofibrosis | 32 (15.8) |
|  | Chronic myeloid leukaemia | 26 (12.8) |
| Median line of cytoreductive therapies | |  |
|  | Actually treated patients | 189 (93.1) |
|  | Hydroxycarbamide | 81 (42.9) |
|  | Pegylated-interferon | 30 (15.9) |
|  | Ruxolitinib | 27 (14.3) |
|  | ABL1 kinase inhibitors | 26 (13.8) |
|  | Anagrelide | 17 (9) |
|  | Pipobroman | 8 (4.1) |
| Tooth cares | Total patients | 39 (19.2) |
|  | Total cares | 44 |
|  | Tooth cracks | 19 (43.2) |
|  | Tooth losses | 13 (29.6) |
|  | Gingival abscesses | 12 (27.3) |

**Supplementary Table 2. Repartition of drugs according to their MPN.**

Peg-Ifn: pegylated-interferon; TKI: tyrosine kinase inhibitors.

**Supplementary Table 3.** **Repartition of oral cares in our population.**

3NEG: triple negative; CALR: calreticulin; JAK2: just another kinase 2; MPL: myeloproliferative leukemia; TKI: tyrosine kinase inhibitors; y: years.
